# *Theileria haneyi*: An Emerging Equids Hemoparasite with Milder Virulence but Major Diagnostic and Therapeutic Implications

**DOI:** 10.3390/pathogens15030309

**Published:** 2026-03-12

**Authors:** Bassma S. M. Elsawy, Heba F. Alzan

**Affiliations:** 1 Parasitology and Animal Diseases Department, Veterinary Research Institute, National Research Centre, Dokki, Giza 12622, Egypt; 2 Tick and Tick-Borne Diseases Research Unit, Veterinary Research Institute, National Research Centre, Dokki, Giza 12622, Egypt; 3 Department of Veterinary Microbiology and Pathology, College of Veterinary Medicine, Washington State University, Pullman, WA 99164, USA

**Keywords:** *Theileria haneyi*, equine piroplasmosis, *T. equi*, genomic divergence, therapeutic resistance, transmission dynamics, tick borne pathogens, competent vector

## Abstract

*Theileria haneyi*, a recently discovered tick-borne hemoparasite infecting equids globally, has significant implications for equine health. Although it is closely related to *T. equi* (sharing 23% genomic divergence), it establishes an asymptomatic carrier state in persistently infected horses, creating a silent transmission reservoir. Its discovery and unique genetics justify its classification as a new taxon. A critical diagnostic challenge is that the lack of the *ema-1* gene in *T. haneyi* prevents its detection by the standard *T. equi* cELISA, emphasizing the need for species-specific tools. Although species-specific PCR assays, including PCR and qPCR targeting genes like *chr1sco* or *ema-11*, respectively, and an indirect ELISA targeting the EMA-11 recombinant protein, have been developed, global genetic variations may limit their serological utility. Therapeutically, *T. haneyi* exhibits resistance to the key antiparasitic drug, imidocarb dipropionate (ID), and interferes with the clearance of co-infecting *T. equi*. Major knowledge gaps persist, particularly regarding the identification of its competent vector. The current work presents an overview of *T. haneyi* virulence, transmission, diagnostics, and therapeutic gaps while pinpointing the deficits in current information necessary for advancing our understanding of the parasite’s biology. Finally, the review discusses and recommends further studies to develop effective control and surveillance strategies for *T. haneyi* infection.

## 1. Introduction

*Theileria* (*T.*) *haneyi* is a recently discovered apicomplexan tick-borne hemoparasite that primarily infects equids, including horses [1] and donkeys [2]. It is closely related to *T. equi*, another causative agent of equine piroplasmosis (EP), a disease with significant global economic implications. It is morphologically smaller than *T. equi* in size. *Theileria haneyi* appears to have a global distribution, with infected equids having been identified in North and South America, Africa [1,3,4,5], China [6,7], and for the first time in donkeys and horses in Egypt by Elsawy et al. [2]. However, it remains unknown whether *T. haneyi* is capable of infecting vertebrate hosts other than equids as *T. equi* which can infect dogs, camels [8,9,10,11], cattle, sheep and goats [12,13]. In the meantime, there is a lack of knowledge regarding its geographical distribution and its prevalence worldwide.

The *T. haneyi* organism causes milder clinical disease (such as variable fever and anemia) than *T. equi* in experimentally infected horses and can be superinfected with *T. equi* [14]. Horses remain persistently infected following the acute stage of the disease, and these asymptomatic horses are presumed to be reservoirs of infectious organisms for competent tick vectors [14].

The global importance of *T. haneyi* to equine health was recently shown by its resistance to imidocarb dipropionate (ID) and its interference with *T. equi* clearance by ID in some co-infected horses. This review shows findings from various academic papers to provide a comprehensive overview of *T. haneyi*, discovery and genomic diversity, virulence and clinical progression, global geographical distribution, treatment efficacy, diagnostic methods progress, and transmission dynamics.

## 2. Data Collection

This study employed a systematic literature search to identify research on *T. haneyi* infections in equids worldwide. The primary search was conducted across PubMed, Scopus, and ScienceDirect, focusing on English-language publications from January 2018 to July 2026.

The search strategy utilized a combination of keywords covering the parasite, tick-borne diseases, virulence, transmission, treatment, diagnosis, and competent vectors. These terms were integrated with animal species (equids, horses, donkeys) and detection methods (microscopical, serological, and molecular examinations), along with geographical filters for global coverage. Boolean operators (“AND” “OR”) were used to construct precise search strings. Supplementary searches were performed on the Egyptian Knowledge Bank website for local journals and Google Scholar to access full-text publications.

## 3. Discovery and Genomic Diversity

*Theileria haneyi* was accidentally discovered in horses at the United States–Mexico border [1]. Phylogenetic analysis based on 18S rDNA revealed that this erythrocyte infective parasite is related to, yet distinct from, other *Theileria* species found in Africa, with its closest relatives being *Theileria* spp. from waterbuck and mountain zebra [1]. The observed sequence variability at the 18S rDNA locus suggests the potential existence of additional cryptic species within the genus [1].

Among the described species, the genome of this novel equine *Theileria* parasite is most similar to that of *T. equi* genotype C. However, the estimated divergence time between *T. haneyi* and *T. equi*, based on genomic sequence data, is over 33 million years [1,15]. The new species, *T. haneyi*, shows a high degree of genetic protein divergence from *T. equi*. This divergence (23%) is even greater than that between two other major, distinct species infecting domestic cattle and African/Asian wild buffalo (*Theileria parva* and *Theileria annulata*, respectively, “18%”) [1]. This significant genomic divergence, coupled with morphological differences, justifies the classification of *T. haneyi* as a new taxon [1].

**Figure 1:**
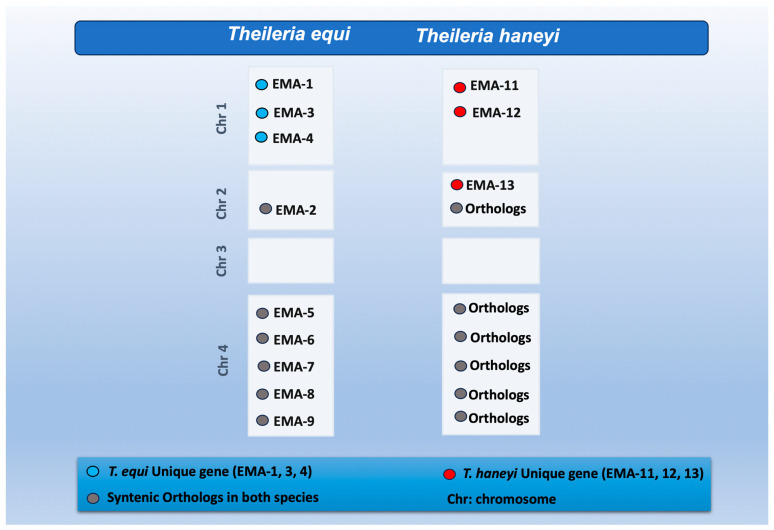
Schematic diagram of comparative EMA genes distribution between *T. equi* and *T. haneyi* genomic chromosomes.

Despite the overall genomic divergence, it was found that *T. haneyi* possesses a nine-member Equi Merozoite Antigen (EMA) superfamily, a multigene family previously thought to be exclusive to *T. equi*. Yet, significant sequence divergence in antigenic loci means that *T. haneyi* is not detectable by currently available diagnostic tests designed for *T. equi* [1]. Briefly, six of the nine *T. equi* EMA genes (*ema-2*, *ema-5* to *ema-9*) have orthologs in *T. haneyi*, but *T. equi*’s *ema-1*, *ema-3*, and *ema-4* (all on chromosome 1) are absent in the latter. This specific deletion of *ema-1* in *T. haneyi* prevents its detection by the *T. equi* cELISA, which targets the EMA-1 protein. In contrast, *T. haneyi* has three unique EMA genes: *ema-11* and *ema-12* on chromosome 1, and *ema-13* on chromosome 2 [1] (Figure 1). This highlights the challenges in apicomplexan parasite surveillance and equine piroplasmosis epidemiological studies, which consequently reflects the importance of developing specific diagnostic tools for the newly identified species *T. haneyi*.

Importantly, although the *T. haneyi* genome has been studied [1], the genome assemblies of *T. haneyi* still have gaps with uncertain chromosome structure, limiting biological interpretation. In a recent study, researchers generated a high-quality, gap-free genome assembly of the *T. haneyi* Eagle Pass isolate using a combination of long- and short-read sequencing technologies. Long-read sequencing enabled the assembly of near-complete chromosomes, while short-read data were used for error correction [16].

## 4. Transmission Dynamics

Understanding the transmission dynamics of *T. haneyi* is crucial for effective disease control. As a tick-borne hemoparasite, its transmission is primarily mediated by ticks. However, identifying competent tick vectors remains an area of ongoing research. A recent study investigated whether *Haemaphysalis longicornis* (*H. longicornis*), also known as the Asian longhorned tick, can transstadially transmit *T. haneyi* to horses or not [17]. This tick species is endemic to East Asia and has spread to other regions, including the USA, where it is known to transmit *Theileria orientalis* Ikeda genotype to cattle [17]. The study found that while *H. longicornis* larvae were efficient at acquiring *T. haneyi* from infected horses; there was no evidence of transstadial transmission to naïve horses [17]. This suggests that *H. longicornis* is not a competent vector for *T. haneyi* transmission, despite its ability to feed efficiently on horses and transmit related *Theileria* species [17]. This finding highlights a significant knowledge gap regarding the natural tick vectors for *T. haneyi*. Further research is needed to identify the specific tick species responsible for transmitting *T. haneyi* across different geographical regions. The ability of *T. haneyi*-infected horses to become asymptomatic carriers also contributes to the complexity of transmission, as these animals can serve as a silent reservoir for the parasite, facilitating its spread without overt clinical signs [14].

It is important to note that the future dissemination of *T. haneyi* is also driven by climate change, expanding tick habitats, and the global movement of asymptomatic equine carriers through trade. Iatrogenic transmission via contaminated veterinary equipment and complex co-infections with other parasites further complicate its dissemination and clinical impact.

## 5. Virulence and Clinical Progression

Initial studies demonstrated reduced clinical severity in spleen-intact horses. Furthermore, it was found that *T. haneyi* is less virulent than *T. equi* in splenectomized horses [14]. *Theileria haneyi* is a less virulent parasite than *T. equi* that can superinfect with *T. equi* in horses; it causes a delayed serological response and is not consistently detected by current *T. equi* diagnostic assays due to limited antigenic cross-reactivity [3]. In early experiments, splenectomized horses survived *T. haneyi* infection and progressed to an asymptomatic carrier state, in stark contrast to the high fatality rate of *T. equi* in splenectomized horses. Thus, it was theorized that *T. haneyi* is less virulent than *T. equi*. This was confirmed by Sears et al. [14], who evaluated clinical data from splenectomized, *T. haneyi*-infected horses and found that seven of the eight splenectomized, *T. haneyi*-infected horses survived and progressed to an asymptomatic carrier state. These data contrast with the high fatality rate observed in *T. equi*-infected splenectomized horses without intermediation [14]. This reduced virulence of *T. haneyi* is probably attributed to its genomic reduction, as its genome is approximately 2 Mbp smaller than that of *T. equi* [14].

In cases of acute *T. haneyi* infection in splenectomized horses, the symptoms included fever (102.4 to 105.8 °F), anemia through decreased packed cell volume (PCV), and variable levels of parasitemia. Parasitemia appeared in waves, with some horses maintaining low levels while others experienced intermittent increases. The ability of *T. haneyi*-infected horses to become asymptomatic carriers has significant implications for disease control and surveillance. These animals can serve as a continuous reservoir for transmission [14], as with *T. equi*, where clinical signs are often subtle without careful observation. However, clinical signs can vary significantly in uncontrolled environments, especially in working or athletic animals [14].

## 6. Diagnostic Methods Progress

**Figure 2:**
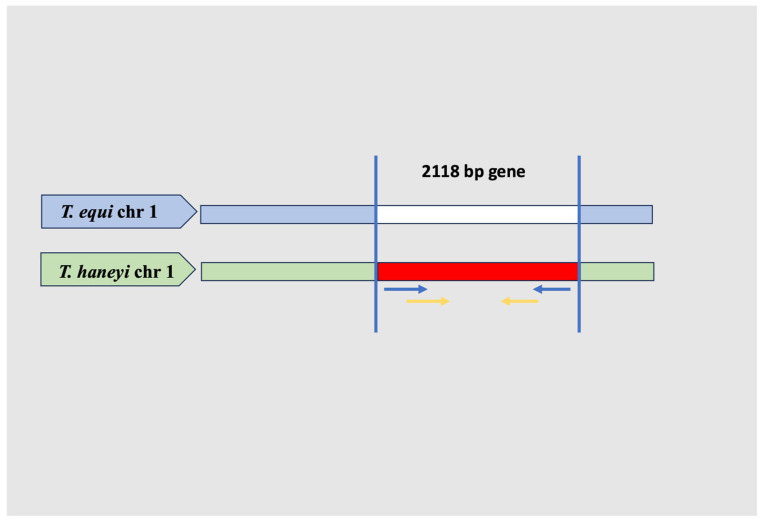
Schematic draws show the nPCR technology for the amplification of the chr1sco gene of *T. haneyi*. The double helix structure of *T. equi* and *T. haneyi* genes was colored light blue and green. The chr1sco gene sequence colored red is *T. haneyi* chromosome, and the missing chr1sco gene sequence of *T. equi* is colored white. The external primers used were labeled with blue arrows, and the internal primers were labeled with orange arrows.

The accurate diagnosis of *T. haneyi* infection is critical for effective management and control, especially given its potential to co-infect with *T. equi* and its resistance to common treatments. Traditional diagnostic methods for equine piroplasmosis, such as nPCR assays targeting *T. equi* genes, have been found to be unreliable for detecting *T. haneyi* due to significant sequence divergence in antigenic loci [1]. To overcome this issue, a gene coding for a protein of unknown function in the syntenic *T. haneyi* locus of the vacated *ema1* gene was selected to detect *T. haneyi* by PCR assay [1,3]. Certain studies used species-specific nested PCR-targeting this gene, which is specific only for *T. haneyi*, to detect its prevalence in countries such as Nigeria (2.7% in horses) [5], South Africa [18] and Egypt (53.1% in horses and 38.1% in donkeys) [2]. In China, Yang et al. [7] used nPCR targeting chromosome 1 single-copy (chr1sco) open reading frame genes, which have no detectable orthologs in *T. equi* or *B. caballi* (Figure 2)*,* for the detection of *T. haneyi* (prevalence 11.7%).

Related studies have developed a new real-time quantitative PCR (qPCR) method based on the *chr1sco* gene, which is considered a straightforward, rapid and sensitive diagnostic method to detect *T. haneyi* [6]. However, false-negative results and a poor correlation between the results of the nested PCR and qPCR assays were reported [6], suggesting that this qPCR assay may not be as specific or as sensitive as the nested PCR in detecting *T. haneyi* infections when based on the *chr1sco* gene. Bhoora et al. [19] targeted the equi merozoite antigen 11 (*Th. ema*-11) gene, which is not found in *T. equi*, as a target for qPCR assays. It has demonstrated efficiency, specificity, and sensitivity in detecting *T. haneyi* *ema-11*. A recent study utilized the *EMA 11* gene and *EMA 10* genes for the detection of *T. haneyi* in Brazil using conventional PCR and semi-nested PCR assays, with a prevalence of 2.1% in an imported horse from Texas, US, and kept in an equestrian center in Brazil [20]. However, the positive sample in the PCR assays targeting the *ema-10* and *ema-11* genes tested negative in the *18S rRNA* and *ema-12*-based PCR, which limited a more precise characterization of the *T. haneyi* detected in that study [20]. Nevertheless, the first detection of *T. haneyi* in Argentina was reported based on *18S rDNA*-based PCR [21].

Recent advancements have focused on developing more specific serological diagnostic tools to detect the presence of antibodies against *T. haneyi* in animal serum. An indirect (i)ELISA based on the equi merozoite antigen 11 (*Th*EMA11) recombinant EMA-11 protein of *T. haneyi* was developed and shown promise in detecting geographically diverse *T. haneyi* strains in the sera of infected horses from different countries around the world [22]. Since the collected samples were from the USA, Germany, Mexico, France, Ireland, Puerto Rico and the Netherlands, there is a risk of potential variation in the EMA-11 protein sequence, which might limit the effectiveness of the indirect ELISA outside of these regions [19]. The same iELISA was utilized in China and showed 16.41% seroprevalence in a cross-sectional analysis of 2627 equine samples [7].

## 7. Global *T. haneyi* Geographical Distribution Analysis

**Figure 3:**
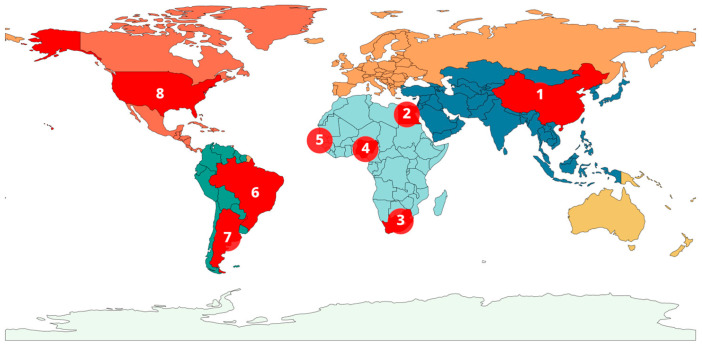
Geographic analysis of *T. haneyi* distribution across various countries. Locations marked in Red (East to West) with Arabic number refers to the country of the published study. 1—China, 2—Egypt, 3—South Africa, 4—Nigeria, 5—Gambia, 6—Brazil, 7—Arganitina and 8—United States of America.

*Theileria haneyi* was first detected in 2018 in a horse at the United States–Mexico border, North America [1]. This initial finding was a result of surveillance efforts, specifically near Eagle Pass, Texas, which led to the parasite sometimes being referred to as the *T. haneyi* (EP) strain [1]. Detection began to be performed in other countries on the Africa continent, such as South Africa [18], Nigeria [5], Gambia [23] and Egypt [2]. Later, it was detected in Asian countries such as China [7], in addition to Brazil and Argentina in South America [20,21]. Current information indicates that *T. haneyi* is distributed across three continents: North America, Africa, and Asia. In Europe, Facile et al. [24] examined equine samples for the presence of *T. haneyi,* targeting the chr1sco gene in nPCR, but all samples were negative. However, there is still a lack of data regarding its distribution in other continents. Even though the presence of *T. haneyi* has been identified on some content, the individual countries have not yet been surveyed within the same continent. This emphasized the need for further surveillance studies in other countries to cover their global range (Table 1 and Figure 3).

## 8. Treatment Efficacy

There is a significant therapeutic gap in the treatment of equine piroplasmosis caused by *T. haneyi*, as evidenced by studies on the effectiveness of common antiparasitic drugs [17] (Table 2). It has been shown that *T. haneyi* is resistant to several widely used therapeutic agents, including imidocarb (ID) dipropionate, buparvaquone, tulathromycin, and diclazuril [25]. Imidocarb dipropionate is often the drug of choice for treating equine piroplasmosis and controlling clinical signs of *T. equi* infection worldwide [25]. However, it fails to treat *T. haneyi*-infected horses. Compounding the issue, *T. haneyi* co-infection can hinder the successful treatment of *T. equi* by ID dipropionate [14]. Thus, this resistance is a significant concern. Additionally, growing concerns regarding ID-resistant parasite strains and their associated toxicity have highlighted the urgent need for novel, safer, and more effective antiparasitic agents.

The in vitro efficacy of tafenoquine succinate (TFQ), a synthetic 8-aminoquinoline with broad antiparasitic activity, against *T. equi* and *B. caballi* as a potential treatment for equine piroplasmosis was tested by Cardillo et al. [26]. However, it has not yet been used for the treatment of *T. haneyi.* It was found that TFQ has potent in vitro activity against *T. equi* and moderate activity against *B. caballi,* along with a mild cytotoxic profile in equine Peripheral Blood Mononuclear Cells (PBMCs). Moreover, certain studies have investigated the temporary efficacy of buparvaquone against *T. haneyi* in chronically infected horses. It was found that the recommended dose of 2.5 mg/kg led to a rapid reduction in *T. haneyi* levels to undetectable levels by nested PCR (nPCR), but the recurrence occurred after a minimum of seven weeks [25]. Subsequent re-administration of buparvaquone at an increased dosage of 6 mg/kg failed to produce a theilericidal effect upon recrudescence [25]. This suggests that buparvaquone may offer only temporary suppression rather than a definitive cure for *T. haneyi*. Accordingly, this complicates treatment strategies for horses infected by *Theileria* spp., highlighting the need for new therapeutic approaches that are effective against *T. haneyi* and do not compromise the treatment of co-existing *T. equi* infections.

## 9. Conclusions and Recommendations

*Theileria haneyi* is a less virulent parasite compared to *T. equi*, yet it poses significant challenges to global equine health due to its drug resistance and ability to establish asymptomatic carrier states. While recent genomic advancements research provides an establishment for understanding its biology, the parasite’s distinct genetic profile from *T. equi* demands the global validation of specific diagnostic tools such as qPCR and nPCR targeting *T. haneyi’s* unique genes, such as the EMA 11 and the chr1sco open reading frame genes.

Future research must spotlight identifying its competent tick vectors through detecting the presence of this parasite in different ticks in the field that infest the farm animals to draw a wider picture for parasite spreading vectors and exploring the associated co-infections across diverse livestock like cattle, camel, sheep and goats to fully interpret its transmission dynamics. Finally, developing targeted therapeutics and vaccines is essential to relieve its impact on equine health and prevent its further spread. The insights gained from studying *T. haneyi* not only contribute to our understanding of equine piroplasmosis but also provide a foundation for defining virulence mechanisms within the broader Apicomplexa phylum, as well as the synergetic effect regarding drug resistance.

## Figures and Tables

**Table 1:** Geographic analysis of *T. haneyi* distribution across various countries.

Continent	Country	Study Type	Salient Findings	Reference
North America	USA/Mexico Border	Surveillance and Discovery	First detection of *T. haneyi* (Eagle Pass strain) in a horse; distinct from *T. equi* based on 18S rDNA.	[1]
Africa	South Africa	Molecular Epidemiology	Identified *T. haneyi* in horses; highlighted the prevalence of co-infections with *T. equi*	[18]
	Nigeria	Molecular Survey	First report in Nigeria; confirmed presence in equine using PCR and sequencing	[5]
	Gambia	Cross-sectional Study	Detection of *T. haneyi* in equids; contributed to the known distribution in West Africa.	[23]
	Egypt	Molecular detection	First molecular evidence of *T. haneyi* in Egyptian horses and donkeys	[2]
Asia	China	Molecular Epidemiology	Report of *T. haneyi* in horses from various regions; showed genetic similarity to the US reference strain.	[7]
South America	Brazil	Molecular Survey	Confirm the presence in Brazilian horse populations; emphasized the risk of silent spread.	[20]
	Argentina	Epidemiological Study	Identified the parasite in horse populations; noted its association with tick-borne pathogens.	[21]
Europe	Italy	Molecular Diagnosis and Identification of Equine Piroplasms	*T. haneyi* was not detected in any of the examined samples	[24]

**Table 2:** Generic and product names of pharmaceutical compounds against *T. haneyi*.

Generic Name	Example Product Name(s)	Observed Efficacy/Status Against *T. haneyi*
Imidocarb dipropionate	Imizol^®^	Resistant/Fails to eliminate the parasite, and its presence can hinder treatment of *T. equi* co-infections.
Buparvaquone	Butalex^®^	Temporary suppression/Initial reduction in parasite levels, but recurrence occurs; higher doses fail upon recrudescence.
Tulathromycin	Draxxin^®^	Resistant/Shown to be ineffective against *T. haneyi* in recent studies.
Diclazuril	Protazil^®^	Resistant/No significant theilericidal effect observed against this species.
Tafenoquine succinate	Arakoda^®^, Krintafel^®^	Untested/Shows potent in vitro activity against *T. equi*, but efficacy against *T. haneyi* is yet to be determined.

## Data Availability

No new data were created or analyzed in this study. Data sharing is not applicable to this article.
